# Association of ultrasound-derived muscle parameters and liver stiffness with low muscle mass in patients with cirrhosis

**DOI:** 10.3389/fmed.2026.1865779

**Published:** 2026-07-08

**Authors:** Yang Cheng, Jiang Lina, Hong Liu, Yu Kang

**Affiliations:** 1 Department of Ultrasound Medicine, Dazhou Second People’s Hospital, Dazhou, China; 2 School of Medicine and Life Sciences, Chengdu University of Traditional Chinese Medicine, Chengdu, China; 3 Department of Ultrasound Medicine, Hospital of Chengdu University of Traditional Chinese Medicine, Chengdu, China

**Keywords:** cirrhosis, high-frequency ultrasound, muscle thickness, sarcopenia, shear wave elastography

## Abstract

**Objective:**

To explore the application value of high-frequency ultrasound combined with two-dimensional shear wave elastography (2D-SWE) in diagnosing low muscle mass in patients with cirrhosis.

**Methods:**

A total of 135 patients with cirrhosis were enrolled in this study. Participants were categorized into a Cirrhosis group (*n* = 77) and a CRS group (*n* = 58) based on the Arm Skeletal Muscle Index (ASMI). Concurrently, 66 age- and sex-matched healthy individuals were recruited as the normal control (NC) group. Analyzed the general characteristics and biochemical parameters of each group, comparing liver elastic modulus (LEM) alongside muscle thickness (MT) and shear wave velocity (SWV) in the right biceps brachii, rectus femoris, and medial head of the gastrocnemius.

**Results:**

Compared with the NC and cirrhosis groups, the CRS group showed lower MT and muscle SWV, but higher LEM (*p* < 0.05). Pearson correlation analysis showed that ASMI was positively correlated with body mass index (BMI), serum albumin levels, MT, and muscle SWV and negatively correlated with age, total bilirubin, LEM, and international normalized ratio (INR) (all *p* < 0.05). Stepwise logistic regression identified MG-SWV, RF-MT, and LEM as factors independently associated with CRS (all *p* < 0.05). The receiver operating characteristic curve (AUC) for CRS revealed the following. Muscle SWV: 0.760–0.820, MT: 0.782–0.878, LEM alone: 0.760, Combined application of muscle parameters and LEM: 0.919 (sensitivity 90.57%, specificity 83.13%) (all *p* < 0.05). The AUC for the combined use of muscle parameters and LEM significantly outperformed that of any single parameter, demonstrating superior diagnostic performance.

**Conclusion:**

Ultrasound measurement of LEM, MT, and muscle SWV offers an effective diagnostic approach for CRS. The combined assessment of muscle parameters with LEM demonstrates enhanced diagnostic accuracy compared to any single parameter.

## 1 Introduction

As the global population ages and chronic diseases become increasingly prevalent, sarcopenia has attracted considerable attention. The Asian Working Group for Sarcopenia (AWGS) defines sarcopenia as age-related progressive skeletal muscle loss accompanied by diminished muscle strength and physical function (1). Cirrhosis, the terminal phase of chronic liver disease, is complicated by sarcopenia in 30–70% of patients, which predicts poor prognosis and complications (2, 3). The pathogenesis of cirrhosis-associated sarcopenia (CRS) involves multiple mechanisms (e.g., abnormal protein metabolism, hyperammonemia, chronic inflammation, hormonal changes, and inactivity) (4–10). Sarcopenia also increases the risk of hospitalization for infections (11). Therefore, routine screening and intervention for sarcopenia in cirrhosis patients are essential to break the vicious cycle and improve outcomes (12).

Early diagnosis of sarcopenia remains challenging (13). Traditional methods (DXA, bioelectrical impedance analysis, CT, MRI) have limitations including radiation, high cost, and poor accessibility (14, 15). Ultrasound has emerged as a non-invasive, real-time, and repeatable tool for muscle assessment, with strong correlation to gold-standard measurements (16–21).

Despite the high prevalence and underdiagnosis of sarcopenia in cirrhosis, ultrasound-measured muscle thickness and shear wave velocity can quantify muscle quantity and quality, and adding liver elastic modulus may capture disease severity, which is closely linked to muscle wasting. However, clinical studies on CRS using ultrasound remain scarce. Becchetti et al. (22) found that 2D-SWE of the rectus femoris was feasible and correlated with frailty. Mahmoud et al. (23) reported that ultrasound-assessed muscle atrophy independently predicted disease severity and poor prognosis. Ciocîrlan et al. (20) showed that rectus abdominis thickness predicted survival. Nevertheless, these studies did not incorporate biochemical markers or liver elastic modulus (LEM), nor did they provide diagnostic thresholds for CRS. Given the rising global incidence of cirrhosis, this study aims to investigate correlations between multiple clinical indicators and CRS, evaluate the diagnostic efficacy of ultrasound (muscle thickness, SWV, and LEM), and establish preliminary diagnostic thresholds for CRS.

## 2 Participants and methods

### 2.1 Study participants

We recruited 135 patients with liver cirrhosis who were admitted to or visited the Infectious Diseases Department of a tertiary hospital in Sichuan, China, between April 2025 and March 2026. Each participant fulfilled the diagnostic criteria established in the “Guidelines for the Diagnosis and Treatment of Liver Cirrhosis” (24). All patients included in this study signed informed consent forms and permitted the reuse of their relevant data. The Institutional Ethics Committees of our hospitals approved this study (No.2026KL-023). Based on the Appendicular Skeletal Muscle Mass Index (ASMI), we categorized the patients into a Cirrhosis group (*n* = 77) and a CRS group (*n* = 58). Concurrently, we enrolled 66 healthy individuals matched for gender and age as the Normal Control (NC) group.

### 2.2 Inclusion and exclusion criteria

#### 2.2.1 Inclusion criteria

Clinically diagnosed cirrhosis for ≥ 5 years; Age 50–70 years; Possession of complete clinical records.

#### 2.2.2 Exclusion criteria

Patients presenting with massive ascites; Severe renal or heart failure accompanied by pitting edema; Patients with various types of cancer; Patients diagnosed with thyroid dysfunction, or pituitary, adrenal, or parathyroid disorders; Musculoskeletal or joint disorders; prolonged bed rest, history of stroke, or lower limb paralysis; Use of medications known to affect muscle metabolism (e.g., antiepileptics, thyroid hormones, growth hormones, glucocorticoids, sex hormones, weight-loss drugs); Recent surgery, severe trauma, severe dehydration, systemic connective tissue disease, or central nervous system disorders; Other chronic conditions such as diabetes mellitus or chronic obstructive pulmonary disease; Presence of cognitive impairment.

### 2.3 Research methods

#### 2.3.1 General data collection

We recorded each subject’s gender, age, height, and weight, and subsequently calculated their body mass index (BMI). Skeletal muscle mass index (ASMI) was precisely measured using dual-energy X-ray absorptiometry (Lunar iDXA). Low muscle mass is defined as an ASMI value of <5.4 kg/m^2^ for females and <7.0 kg/m^2^ for males (1).

#### 2.3.2 Laboratory data collection

Following a standardized 10–12 h overnight fast, venous blood samples were collected from all subjects the subsequent morning. All assays were conducted on fully automated biochemical and coagulation analyzers. Human serum albumin levels were quantified using the bromocresol green method, transaminase levels were measured by the rate method, and International Normalized Ratio (INR) values were determined through coagulation testing followed by formula calculation.

#### 2.3.3 Ultrasound data acquisition

The examination was carried out in a quiet environment at a room temperature of 25 degrees. Muscle parameters were obtained via Mindray Resona R9 color Doppler ultrasound with a 3–14 MHz linear array probe, targeting the right rectus femoris (RF), gastrocnemius medial head (MG), and biceps brachii (BB). Key parameters: shear wave velocity (SWV, via 2D-SWE in relaxed muscle) and muscle thickness (MT, via high-frequency ultrasound), which provide reliable evidence of sarcopenia (25). MT Measurement Procedure: Biceps Brachii: Examinee supine, rested 5–8 min; right forearm extended, palm pronated; measure max thickness at mid-upper arm. Rectus Femoris: Supine legs extended; measure max thickness at the mid-thigh’s thickest point. Gastrocnemius: Prone, feet suspended, arms crossed; measure max thickness at the medial head’s thickest point. Elasticity Imaging Protocol (Rectus Femoris as example): After MT measurement, rotate the probe 90° to align with the muscle’s long axis, switch to SWE mode. Place a 3 mm sampling circle at the midpoint of the max thickness ( ≥ 5 mm from bone/fascia), avoiding pressure. Freeze image at 100% confidence to record SWV; measure 5 times ( ≥ 3 s intervals), take median. Same protocol for BB and MG SWV measurements.

Ultrasound assessment of liver stiffness: Utilizing 2D-SWE technology, LEM (liver stiffness) values (in kPa) were acquired during patient breath-hold with a Mindray Resona R9 color Doppler ultrasound system equipped with a convex array probe (1–6 MHz frequency range). Following a 4–6-h or longer fast, patients lie supine on the examination bed, with the right arm raised and positioned against the forehead or bed surface. Scanning occurred at the right anterior axillary line within the 7th–9th intercostal space. The probe maintained a perpendicular alignment with the skin, carefully avoiding blood vessels, and the sampling frame was positioned >2 cm from the liver capsule. The median value from five valid measurements was recorded as the result.

**FIGURE 1:**
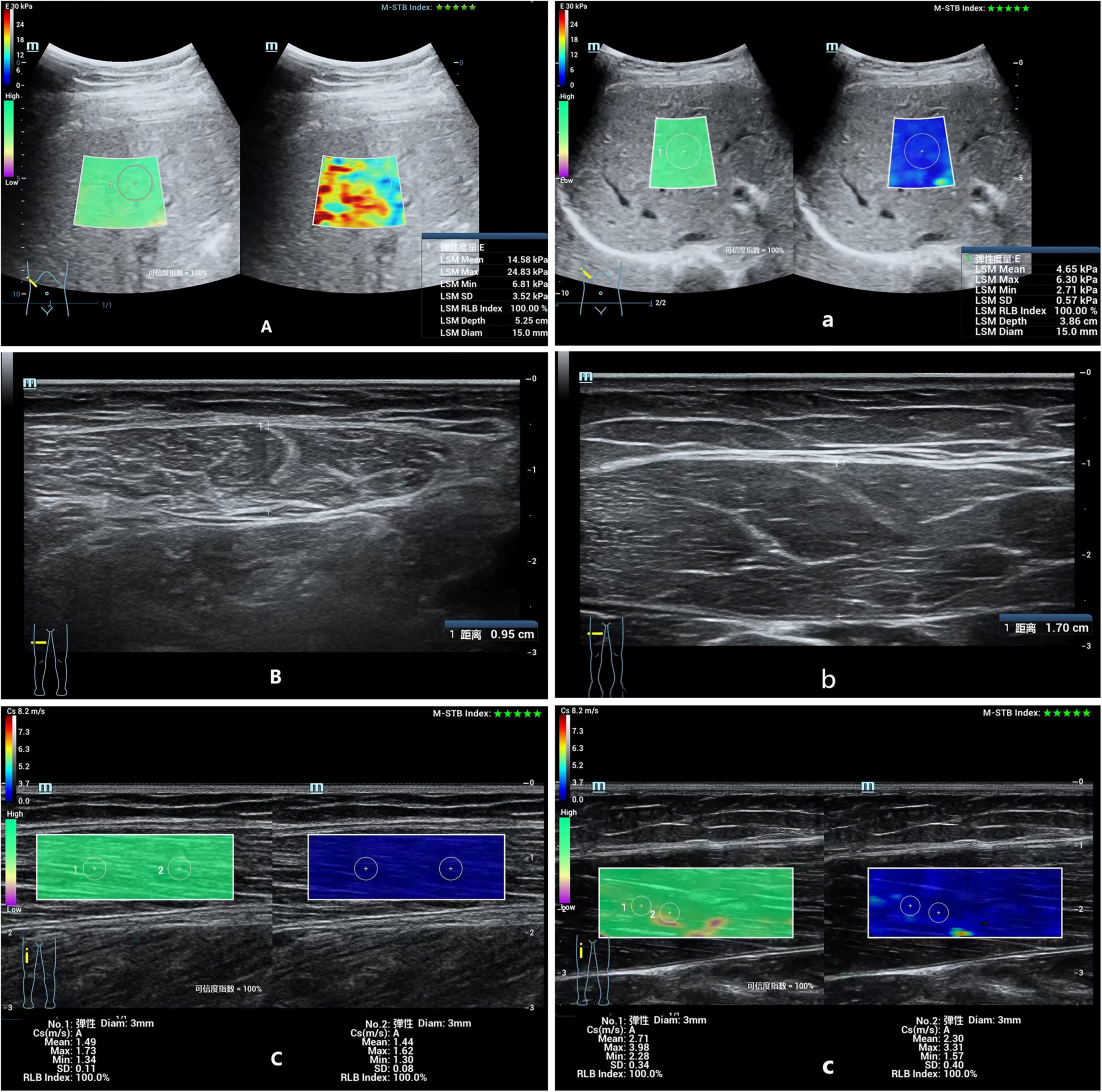
Comparison of selected ultrasound parameters between patients with CRS and healthy individuals. Capital letters denote patients with CRS. Lowercase letters denote healthy individuals. **(A)** Liver elastic modulus, **(B)** rectus femoris thickness, **(C)** rectus femoris shear wave velocity. **(a)** Liver elastic modulus, **(b)** rectus femoris thickness, **(c)** rectus femoris shear wave velocity. CRS, Cirrhosis-Related Sarcopenia.

A Physician with expertise in musculoskeletal ultrasound performs the above examination procedures (ultrasound examination example image, Figure 1).

Reproducibility assessment: Before the formal study, a reproducibility test was conducted on 7 volunteers. Each volunteer underwent ultrasound examinations on three consecutive days, with 5 repeated measurements per day. The intra-observer intraclass correlation coefficient (ICC) for MT was 0.94 (95% CI: 0.89–0.97), and for SWV was 0.91 (95% CI: 0.85–0.95). The coefficient of variation (CV%) was 6.1%. These results indicate good intra-observer reliability.

#### 2.3.4 Statistical analysis methods

Data were analyzed using SPSS version 28.0. Categorical data are expressed as *n* (%) and were compared using the chi-square test. Normally distributed continuous data are presented as mean ± standard deviation ($\bar{x}$ ± s); pairwise comparisons were performed using the *t*-test, and multiple group comparisons using one-way ANOVA. Pearson correlation analysis explored the relationship between ASMI and various indicators.

Multicollinearity diagnosis: Before logistic regression, variance inflation factors (VIFs) were calculated for all candidate independent variables (RF-SWV, RF-MT, MG-SWV, MG-MT, BB-SWV, BB-MT, LEM, BMI, albumin, age, total bilirubin, INR). A VIF > 5 was considered indicative of significant collinearity. For the sensitivity analysis (adjusting for albumin, total bilirubin, and INR), variance inflation factors (VIFs) were also calculated to assess multicollinearity among the included variables (LEM, albumin, total bilirubin, and INR).

Binary logistic regression with stepwise selection: Forward stepwise logistic regression (entry *P* < 0.05, removal *P* > 0.10) was performed to identify factors independently associated with CRS, with “presence of sarcopenia” (CRS group vs. cirrhosis group) as the dependent variable.

Internal validation of the final model: 10-fold cross-validation (repeated 100 times) was used to assess overfitting, and the mean area under the curve (AUC) of the validation sets was reported. Model calibration was evaluated using bootstrap resampling (1,000 iterations), and the calibration slope and intercept were calculated. A *P* < 0.05 was considered statistically significant.

Comparison of ROC curves: Pairwise comparisons of AUCs were limited to three pre-specified comparisons based on the primary hypothesis that the combined model outperforms any single parameter: combined model vs. RF-MT, combined model vs. MG-SWV, and combined model vs. LEM. DeLong’s test for two correlated ROC curves (pROC package in R, method = “delong”) was used. Because these comparisons were hypothesis-driven and limited in number, no adjustment for multiple comparisons was applied. A two-sided *P* < 0.05 was considered statistically significant.

## 3 Results

### 3.1 Comparative analysis of general characteristics, biochemical indicators, and ultrasound measurement parameters across groups

The CRS group demonstrated lower BMI and ASMI compared to the other two groups (*P* < 0.05). Liver stiffness was elevated in both the CRS and Cirrhosis groups compared to the NC group (*P* < 0.05), though no significant disparity emerged between the CRS and Cirrhosis groups. Muscle parameters (MT and SWV) were lower in the CRS group compared to the other two groups (*P* < 0.05). Most muscle parameters showed no notable difference between the Cirrhosis and NC groups. Albumin levels reached their lowest point in the CRS group, while INR and total bilirubin peaked in this same group. Alanine Aminotransferase (ALT) levels, Aspartate Aminotransferase (AST) levels, and the AST/ALT ratio were higher in the CRS and Cirrhosis groups compared to the NC group (*P* < 0.05); however, no significant difference was observed between the CRS and Cirrhosis groups (Table 1).

**TABLE 1:** Comparative analysis of general characteristics, biochemical indicators, and ultrasound measurement parameters across groups.

Group	Number of cases (n)	Gender (male/female)	Age (years)	BMI (kg/m^2^)	ASMI (kg/m^2^)	LEM (kPa)	RF-SWV (m/s)	RF-MT (mm)	MG-SWV (m/s)	MG-MT (mm)
CRS	58	37 (62.8) 21 (36.2)	62.1 ± 6.6b	20.1 ± 2.1a	5.5 ± 0.8a	18.46 ± 5.24a	1.45 ± 0.23a	13.3 ± 1.3a	1.51 ± 0.25a	12.7 ± 1.5a
Cirrhosis	77	41 (53.2) 36 (46.8)	61.5 ± 7.1b	22.3 ± 3.1b	7.2 ± 1.0b	15.82 ± 3.75b	2.21 ± 0.25b	19.7 ± 3.5b	2.15 ± 0.34b	18.6 ± 1.9b
NC	66	37 (56.9) 28 (43.1)	63.3 ± 5.7b	23.8 ± 3.7c	8.1 ± 1.1c	5.85 ± 1.19c	2.32 ± 0.33c	20.3 ± 4.7c	2.34 ± 0.26c	19.4 ± 2.1c
Group	Number of cases (n)	BB-SWV (m/s)	BB- MT (mm)	Albumin (g/L)	Total Bilirubin (μmoL/L)	INR	ALT (U/L)	AST (U/L)	AST/ALT	
CRS	58	1.41 ± 0.27 a	12.4 ± 1.1 a	36.1 ± 5.5a	22.3 ± 12.8b	1.2 ± 0.3a	48 ± 22b	67 ± 26b	1.40 ± 0.31b	
Cirrhosis	77	2.13 ± 0.21b	18.3 ± 2.3b	41.1 ± 6.1b	18.9 ± 11.7b	1.1 ± 0.2b	45 ± 21b	63 ± 25b	1.32 ± 0.25b	
NC	66	2.25 ± 0.32c	18.7 ± 3.3c	44.2 ± 7.3c	18.3 ± 11.1b	1.0 ± 0.1b	27 ± 16a	31 ± 13a	1.05 ± 0.17a	

Data are n (%), mean ± SD. Identical letters indicate no difference between groups; different letters indicate differences, *p*<0.05. BMI, body mass index; ASMI, skeletal muscle mass index; LEM, liver elastic modulus; RF-SWV, rectus femoris shear wave velocity; RF-MT, rectus femoris thickness; MG-SWV, gastrocnemius medial head shear wave velocity; MG-MT, gastrocnemius medial head thickness; BB-SWV, biceps brachii shear wave velocity; BB-MT, biceps brachii thickness.

### 3.2 Correlation between ASMI and other clinical indicators

Pearson correlation analysis linking ASMI with multiple variables revealed: ASMI exhibited strong positive correlations with muscle SWV (*r* = 0.641–0.722, *P* < 0.001) and MT (*r* = 0.602–0.637, *P* < 0.001), alongside BMI (*r* = 0.417, *P* < 0.05) and albumin (*r* = 0.453, *P* < 0.05). Conversely, ASMI displayed a significant negative correlation with liver elastic modules (*r* = −0.593, *P* < 0.001), along with weak to moderate negative associations with age (*r* = −0.461, *P* < 0.05), total bilirubin (*r* = −0.353, *P* < 0.05), and INR (*r* = −0.321, *P* < 0.05) (Table 2).

**TABLE 2:** Correlation between ASMI and various indicators (r).

Indicator	Statistic	RF-SWV	RF-MT	MG-SWV	MG-MT	BB-SWV	BB-MT	LEM	BMI
ASMI	*r*	0.658	0.613	0.722	0.637	0.641	0.602	−0.593	0.417
	*P*	< 0.001	<0.001	< 0.001	<0.001	< 0.001	<0.001	< 0.001	0.002
	Albumin	INR value	Age	Total Bilirubin	ALT	AST	AST/ALT		
ASMI	*r*	0.453	−0.321	−0.461	−0.353	−0.283	−0.364	−0.349	
	*P*	0.017	0.031	0.025	0.042	0.236	0.385	0.095	

RF-SWV, rectus femoris shear wave velocity; RF-MT, rectus femoris thickness; MG-SWV, gastrocnemius medial head shear wave velocity; MG-MT, gastrocnemius medial head thickness; BB-SWV, biceps brachii shear wave velocity; BB-MT, biceps brachii thickness; LEM, liver elastic modulus; BMI, body mass index.

### 3.3 Multicollinearity diagnosis and stepwise logistic regression analysis

The VIF values among candidate variables showed moderate to high collinearity between several muscle parameters: RF-SWV and MG-SWV (VIF = 5.13), and RF-MT and BB-MT (VIF = 4.82). All other VIFs were < 3.

Forward stepwise logistic regression was therefore applied to select independent factors for CRS. The final model retained MG-SWV, RF-MT, and LEM (Table 3). All three variables had VIF values < 3, indicating no remaining significant collinearity. MG-SWV (OR = 0.024, 95% CI: 0.013–0.044) and RF-MT (OR = 0.546, 95% CI: 0.444–0.671) were protective factors, while LEM (OR = 1.200, 95% CI: 1.086–1.326) was a risk factor (all *P* < 0.01). The events per variable (EPV) for the final model was 58/3 ≈ 19.3, well above the recommended minimum of 10.

**TABLE 3:** Stepwise logistic regression analysis of factors associated with CRS.

Variable	β	*P*	Exp (β)	Lower bound of 95% confidence interval for EXP(β)	
				Lower bound	Upper bound
RF-MT	−0.605	0.004	0.546	0.444	0.671
MG-SWV	−3.733	< 0.001	0.024	0.013	0.044
LEM	0.182	0.002	1.200	1.086	1.326

RF-SWV, rectus femoris shear wave velocity; RF-MT, rectus femoris thickness; MG-SWV, gastrocnemius medial head shear wave velocity; MG-MT, gastrocnemius medial head thickness; BB-SWV, biceps brachii shear wave velocity; BB-MT, biceps brachii thickness; LEM, liver elastic modulus.

### 3.4 Internal validation of the combined model

Based on the three variables selected by stepwise regression (MG-SWV, RF-MT, LEM), the predicted probability of CRS was calculated. The AUC of this combined model was 0.919 (95% CI: 0.863–0.975). Ten-fold cross-validation yielded a mean validation AUC of 0.896 (95% CI: 0.841–0.951), which was only 0.023 lower than the training-set AUC, suggesting a low risk of overfitting. Bootstrap calibration showed a slope of 0.94 (ideal = 1) and an intercept of 0.03 (ideal = 0), indicating good calibration.

### 3.5 Diagnostic performance of muscle parameters and LEM for CRS

**FIGURE 2:**
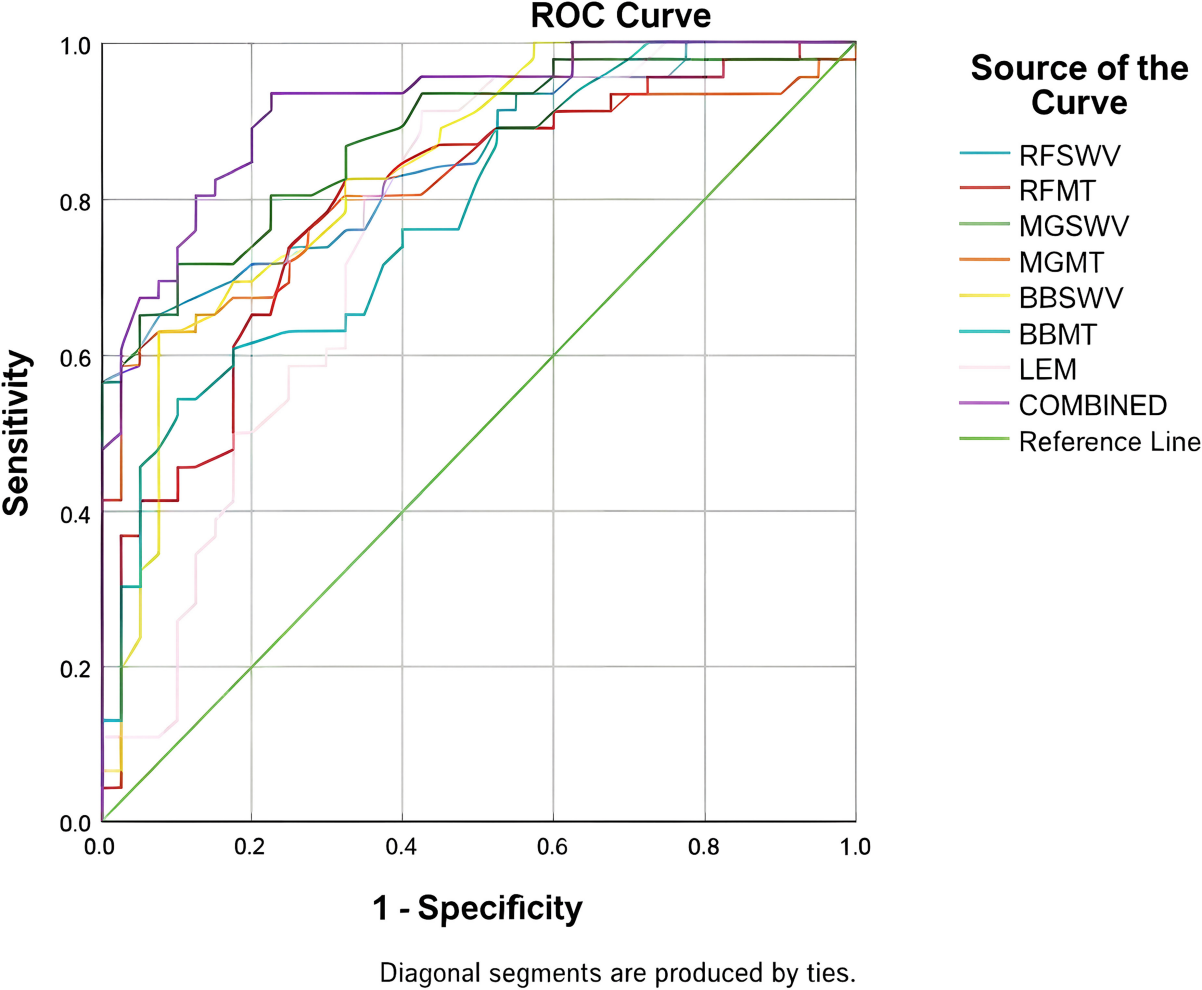
ROC curves for predicting CRS using muscle thickness, muscle shear wave velocity, liver elastic modulus alone, and muscle parameters combined with liver elastic modulus. RF-SWV, rectus femoris shear wave velocity; RF-MT, rectus femoris thickness; MG-SWV, gastrocnemius medial head shear wave velocity; MG-MT, gastrocnemius medial head thickness; BB-SWV, biceps brachii shear wave velocity; BB-MT, biceps brachii thickness; LEM, liver elastic modulus.

The receiver operating characteristic curve (AUC) and optimal diagnostic thresholds for CRS were as follows. RF-SWV: 0.848, 1.62 m/s (sensitivity 83.77%, specificity 81.38%), *p* < 0.001; RF-MT: 0.795, 15.3 mm (sensitivity 78.21%, specificity 72.47%), *p* < 0.05; MG-SWV: 0.878, 1.67 m/s (sensitivity 86.62%, specificity 82.41%), *p* < 0.001; MG-MT: 0.820, 14.5 mm (sensitivity 80.47%, specificity 75.63%), *p* < 0.001; BB-SWV: 0.836, 1.51 m/s (sensitivity 81.33%, specificity 76.59%), *p* < 0.001; BB-MT: 0.782, 13.7 mm (sensitivity 76.69%, specificity 71.15%), *p* < 0.05; LEM alone: 0.760, 18.33 kPa (sensitivity 74.11%, specificity 70.06%), *p* < 0.05; Combined application of muscle parameters and LEM: 0.919 (sensitivity 90.57%, specificity 83.13%), *p* < 0.001. The AUC for the combined use of muscle parameters and LEM significantly outperformed that of any single parameter, demonstrating superior diagnostic performance (Figure 2 and Table 4).

**TABLE 4:** ROC curve analysis of ultrasound single and combined parameters for evaluating CRS.

Parameter	Threshold	AUC value	95% CI	Sensitivity (%)	Specificity (%)	*P*
RF-SWV	1.62 m/s	0.848	0.769–0.927	83.77	81.38	<0.001
RF-MT	15.3 mm	0.795	0.699–0.890	78.21	72.47	0.002
MG-SWV	1.67 m/s	0.878	0.806–0.949	86.62	82.41	<0.001
MG-MT	14.5 mm	0.820	0.730–0.910	80.47	75.63	0.001
BB-SWV	1.51 m/s	0.836	0.750–0.921	81.33	76.59	<0.001
BB-MT	13.7 mm	0.782	0.687–0.877	76.69	71.15	0.025
LEM	18.33 kPa	0.760	0.654–0.865	74.11	70.06	0.013
Combined		0.919	0.863–0.975	90.57	83.13	<0.001

The combined model is based on the predicted probability derived from the stepwise logistic regression model (MG-SWV, RF-MT, LEM) shown in Table 3. F-SWV, rectus femoris shear wave velocity; RF-MT, rectus femoris thickness; MG-SWV, gastrocnemius medial head shear wave velocity; MG-MT, gastrocnemius medial head thickness; BB-SWV, biceps brachii shear wave velocity; BB-MT, biceps brachii thickness; LEM, liver elastic.

### 3.6 Pairwise comparison of AUCs using DeLong’s test

DeLong’s test for the three pre-specified comparisons showed that the combined model (AUC = 0.919) had a significantly higher AUC than RF-MT alone (AUC = 0.795, *Z* = 2.65, *P* = 0.008), MG-SWV alone (AUC = 0.878, *Z* = 2.36, *P* = 0.018), and LEM alone (AUC = 0.760, *Z* = 2.97, *P* = 0.003). No adjustment for multiple comparisons was applied as these comparisons were limited and hypothesis-driven (see Methods) (Table 5).

**TABLE 5:** Pairwise comparison of AUCs using DeLong’s test.

Comparison	AUC1	AUC2	ΔAUC	*Z*	*P*-value
Combined vs. MG-SWV	0.919	0.878	0.041	2.36	0.018
Combined vs. RF-MT	0.919	0.795	0.124	2.65	0.008
Combined vs. LEM	0.919	0.760	0.159	2.97	0.003

The combined model is based on the predicted probability from the stepwise logistic regression model (MG-SWV, RF-MT, LEM) shown in Table 3. Only three pre-specified comparisons were performed (combined model vs. each individual parameter). Because these comparisons were hypothesis-driven and limited in number, no adjustment for multiple comparisons was applied. All *P*-values are derived from DeLong’s test for two correlated ROC curves. RF-MT, rectus femoris thickness; MG-SWV, gastrocnemius medial head shear wave velocity; LEM, liver elastic modulus. RF-MT, rectus femoris thickness; MG-SWV, gastrocnemius medial head shear wave velocity; LEM, liver elastic modulus.

### 3.7 Sensitivity analysis adjusting for liver function parameters

To examine whether the association between LEM and CRS is independent of liver function, we added albumin, total bilirubin, and INR (the core components of the MELD score) as covariates to the stepwise logistic regression model. Multicollinearity diagnosis after adding these covariates showed that all VIFs were below 5 (LEM: VIF = 2.31, albumin: VIF = 1.94, total bilirubin: VIF = 2.05, INR: VIF = 1.87), indicating no significant collinearity. After adjustment for these three parameters, LEM remained significantly associated with CRS (OR = 1.12, 95% CI: 1.01–1.25, *P* = 0.031), indicating that LEM provides incremental predictive value beyond conventional liver function indicators.

## 4 Discussion

Compared with other studies (20, 22, 23, 26–29), this research did not limit itself to a single muscle metric for ultrasound applications in secondary sarcopenia associated with chronic diseases. Instead, it incorporated multiple muscle metrics from both the upper and lower limbs, along with specific indicators of CRS development, such as liver function and liver stiffness. This study comprehensively incorporates ultrasound measurement parameters and laboratory indicators for CRS assessment.

Cross-group comparative analysis revealed that patients in the CRS group exhibited lower BMI, ASMI, MT, and muscle SWV, along with decreased serum albumin levels and elevated ALT, AST, AST/ALT ratio, INR, and total bilirubin levels. Regarding the matching strategy for the NC group, only age and sex were matched, while BMI and nutritional status (albumin) were intentionally not matched between the NC and CRS groups. The CRS group was expected to have lower BMI and albumin levels due to the inherent pathophysiological characteristics of cirrhosis-related sarcopenia, including malnutrition and altered body composition. Therefore, these differences are intrinsic to the disease phenotype rather than a flaw in the matching design. The age- and sex-matched design minimized confounding by the two strongest factors affecting muscle mass, allowing a clearer demonstration of the muscle parameter differences attributable to CRS itself. The synchronous alterations in CRS group muscle parameters and liver function biomarkers further elucidate the close pathophysiological association between sarcopenia and liver dysfunction, underscoring the necessity for integrated assessment of these parameters in comprehensive CRS evaluation. Significant intergroup differences in MT values and muscle SWV values provide crucial reference points for establishing optimal diagnostic thresholds.

Pearson’s correlation analysis revealed no significant association between transaminases and ASMI. Age and INR, along with total bilirubin, exhibited moderate negative correlations with ASMI, whereas BMI and albumin demonstrated moderate positive correlations with ASMI. These results indicate that ASMI exhibits stronger associations with nutritional status, body composition, and systemic liver function than with markers of hepatocellular injury. The negative correlation between age and ASMI corroborates the established age-related sarcopenia pattern, whilst the positive correlation with albumin underscores nutrition’s pivotal role in maintaining muscle mass—particularly for liver cirrhosis patients prone to malnutrition. The negative correlations with bilirubin and INR may reflect the impact of liver impairment on muscle metabolism: elevated bilirubin in advanced disease disrupts protein synthesis, and INR abnormalities (indicating coagulation dysfunction) may reflect metabolic disturbances affecting muscle health. Conversely, no significant correlation was observed between transaminases and ASMI, indicating no direct relationship between fluctuations in liver enzymes and changes in ASMI. These findings provide crucial insights into the determinants of muscle mass in liver disease, revealing potential targets for interventions to preserve muscle health and improve patient outcomes.

Pearson correlation analysis revealed that MT and muscle SWV were significantly positively correlated with ASMI, whereas LEM was strongly negatively correlated with ASMI. Binary logistic regression analysis indicated that MT and muscle SWV measurements constituted protective factors for CRS, while LEM represented a risk factor. ROC curve analysis confirmed that MT and muscle SWV possess good diagnostic value for sarcopenia. Furthermore, combining muscle parameters with LEM significantly enhances diagnostic accuracy (AUC 0.919, Sensitivity 90.57%, Specificity 83.13%). Establishing diagnostic thresholds for MT, LEM, and muscle SWV in CRS facilitates its early detection. Advances in ultrasound elastography now enable direct observation of muscle structure and function. This capability allows prediction of sarcopenia even before morphological changes appear, thereby facilitating crucial early diagnosis and intervention (26). Our findings show that quantifying MT and muscle SWV with ultrasound in cirrhotic patients captures critical changes in muscle mass: reduced muscle SWV and MT reflect lower muscle mass. Both parameters correlate with declining muscle mass. Concurrently, 2D-SWE accurately measures liver stiffness in cirrhotic patients, and robust studies confirm its high precision in detecting liver fibrosis and cirrhosis (27). By measuring the LEM with 2D-SWE, we investigated the correlation between liver stiffness and muscle mass in patients with cirrhosis. Liver stiffness is negatively correlated with muscle mass. It should be noted that the worsening of cirrhosis, malnutrition, systemic inflammation, metabolic dysfunction, and reduced physical activity collectively led to increased liver stiffness and a decline in muscle mass. It is therefore possible that the observed correlation primarily reflects an association with disease severity, rather than a direct biomechanical interaction between the liver and the muscles. This study systematically evaluates the diagnostic efficacy of ultrasound for CRS, establishing a comprehensive, multidimensional assessment framework by integrating muscle mass parameters with liver stiffness parameters.

Sensitivity analysis adjusting for albumin, total bilirubin, and INR confirmed that LEM remained independently associated with CRS, suggesting that its predictive value is not solely explained by these routine liver function markers.

The ultrasound protocol proposed in this study (measuring MG-SWV, RF-MT, and LEM) is non-invasive, radiation-free, and can be completed at the bedside within 15 min. Compared with CT or MRI, ultrasound is more readily available and cost-effective in most clinical settings. However, it requires an initial investment in shear-wave elastography equipment and staff training. Implementing this protocol in routine hepatology practice could facilitate the early detection of sarcopenia, thereby enabling timely nutritional and exercise interventions. A cost-effectiveness analysis will be necessary in the future.

This study has several limitations that should be acknowledged. Due to high correlations among ultrasound parameters (VIF > 5), stepwise regression was used to select MG-SWV, RF-MT, and LEM, but this approach is exploratory. Internal cross-validation and bootstrap suggested low overfitting (AUC decrease 0.023, slope 0.94), yet they cannot replace external validation. The cut-off values were derived from a single Chinese center and lack external validation; absolute SWV/MT values depend on equipment, probe, operator, positioning, etc., so caution and local validation are needed. Muscle SWV is activation-dependent, but we did not objectively verify relaxation. Intra-observer reliability was good (ICC ≥ 0.91, CV 6.1%); inter-observer reliability was not assessed. The observed muscle changes are not cirrhosis-specific. Child-Pugh and MELD scores were not recorded, limiting adjustment for liver disease severity. However, after adjusting for albumin, bilirubin, and INR (MELD components) in stepwise regression, LEM remained independently associated with CRS. Other ultrasound parameters (e.g., fascicle length, pennation angle, cross-sectional area) and serum biomarkers of muscle metabolism (e.g., myostatin, irisin) were not analyzed. Additionally, the AWGS-based ASMI cut-offs used in this study are population-specific for Asian populations, which may limit the generalizability of our findings to other ethnic groups. Future multicenter, externally validated studies with standardized protocols are required.

## 5 Conclusion

In summary, high-frequency ultrasound combined with 2D-SWE demonstrates diagnostic value for CRS in this single-center setting, consistent with previous research findings on the value of ultrasound in chronic disease-related sarcopenia (29–32). Stepwise logistic regression identified MG-SWV, RF-MT, and LEM as factors independently associated with CRS. The combined model using these three variables showed good performance. The proposed cut-off value may serve as a preliminary reference. However, these findings are derived from a single-center sample without external validation; their generalization to other populations or different ultrasound systems should be made with caution. External validation in diverse populations is required before clinical application.

## Data Availability

The raw data supporting the conclusions of this article will be made available by the authors, without undue reservation.
